# The Impact of Same-Day Antiretroviral Therapy Initiation Under the World Health Organization Treat-All Policy

**DOI:** 10.1093/aje/kwab032

**Published:** 2021-02-12

**Authors:** Bernhard Kerschberger, Andrew Boulle, Rudo Kuwengwa, Iza Ciglenecki, Michael Schomaker

**Keywords:** Eswatini, HIV, rapid ART, same-day ART, TMLE, treat-all

## Abstract

Rapid initiation of antiretroviral therapy (ART) is recommended for people living with human immunodeficiency virus (HIV), with the option to start treatment on the day of diagnosis (same-day ART). However, the effect of same-day ART remains unknown in realistic public sector settings. We established a cohort of ≥16-year-old patients who initiated first-line ART under a treat-all policy in Nhlangano (Eswatini) during 2014–2016, either on the day of HIV care enrollment (same-day ART) or 1–14 days thereafter (early ART). Directed acyclic graphs, flexible parametric survival analysis, and targeted maximum likelihood estimation (TMLE) were used to estimate the effect of same-day-ART initiation on a composite unfavorable treatment outcome (loss to follow-up, death, viral failure, treatment switch). Of 1,328 patients, 839 (63.2%) initiated same-day ART. The adjusted hazard ratio of the unfavorable outcome was higher, 1.48 (95% confidence interval: 1.16, 1.89), for same-day ART compared with early ART. TMLE suggested that after 1 year, 28.9% of patients would experience the unfavorable outcome under same-day ART compared with 21.2% under early ART (difference: 7.7%; 1.3%–14.1%). This estimate was driven by loss to follow-up and varied over time, with a higher hazard during the first year after HIV care enrollment and a similar hazard thereafter. We found an increased risk with same-day ART. A limitation was that possible silent transfers that were not captured.

## Abbreviations

ART: antiretroviral therapy
CI: confidence interval
HIV: human immunodeficiency virus
LTFU: loss to follow-up
TMLE: targeted maximum likelihood estimation

The World Health Organization “treat-all” policy recommends lifelong antiretroviral therapy (ART) at the time of diagnosis for all people living with human immunodeficiency virus (HIV), irrespective of immunological criteria (1). Despite high uptake of this policy in Africa (2), of 20.6 million people living with HIV in Eastern and Southern Africa, treatment coverage (67%) and viral suppression (58%) remained below the Joint United Nations Programme on HIV/AIDS targets in 2018, with an additional 3.0 million people living with HIV needing to access treatment and achieve viral suppression (3, 4).

Accelerated ART initiation has been proposed to overcome some of these gaps (5, 6). A systematic review found that ART initiation on the same day as HIV diagnosis or the day of treatment eligibility improved treatment uptake, HIV care retention, and viral suppression (7). Based on this evidence, the World Health Organization released guidelines in 2017 recommending ART initiation within 7 days of HIV diagnosis (rapid ART), with the possibility of initiating treatment on the same day as HIV diagnosis (same-day ART) for patients ready to start (8).

Because HIV programs allow for accelerated ART initiation under the treat-all policy, and most treatment initiations already occur quickly (within 14–30 days after HIV diagnosis or care enrollment (9–13)), the question increasingly shifts to how much more rapidly ART can be initiated in routine resource-limited settings. This question has also been raised recently in public HIV treatment programs in high-income countries (14). Concerns were specifically raised about the feasibility of same-day ART initiation in realistic public sector settings because of lack of real-world evidence and practical limitations. First, evidence of the benefits of accelerated ART mainly originated from randomized trials (7). These trials often applied additional procedures not routinely available in resource-limited settings (e.g., accelerated counseling protocols, treatment readiness survey), used treatment eligibility criteria in use before the treat-all policy, restricted ART interventions to specific patient groups (e.g., nonpregnant adults) or few facilities, or applied different definitions of same-day ART (7, 15–17). In contrast, benefits of same-day ART initiation remained uncertain in observational studies (7). Second, real-world effectiveness might be compromised because of preexisting constraints in the public sector, such as resource limitation (e.g., human resources), overburdened health facilities, and suboptimal quality of care (18–21).

The treat-all policy has been implemented in a public-sector setting in southern Eswatini (formerly Swaziland) since 2014, with same-day ART initiation increasingly practiced (12). Therefore, this setting provides a unique opportunity to better understand how much more quickly ART should be started in a context where it is already started quickly. We aimed to answer the following questions: 1) how is same-day ART being implemented in a public-sector program applying the treat-all approach, and 2) what is the effect of same-day ART initiation compared with early ART initiation (1–14 days after HIV care enrollment) on treatment outcomes for patients starting treatment quickly.

## METHODS

### Setting

Details of the study setting have been described elsewhere (12, 22). In brief, Eswatini has an HIV prevalence of 32% among adults aged 18–49 years, and annual tuberculosis incidence was 308 cases per 100,000 population with 75% HIV coinfection in 2017 (23, 24). The treat-all policy was piloted in 8 primary-care and 1 secondary-care public-sector facilities in the predominantly rural Nhlangano health zone of the Shiselweni region. Other facilities of the region were excluded from this study because they applied the CD4 350 and 500 cells/mm^3^ treatment eligibility thresholds as recommended by national treatment guidelines (12). ART initiation was possible in the absence of baseline CD4 cell counts and biochemistry results (25). ART initiation on the day of facility-based HIV care enrollment was policy for pregnant/lactating women and encouraged for other patients in the absence of (presumptive) opportunistic infections (25, 26). Without specific standard operating procedures in place for same-day ART initiation under the treat-all policy at that time, the clinician decided on the timing of ART initiation after clinical and psychological readiness assessment, the patient’s perceived readiness, and other clinical considerations. Because HIV care registration and ART initiation were performed by facility-based clinicians, same-day ART initiation (on the day of HIV diagnosis) was in practice infeasible for HIV-positive patients transferred in from non-HIV care facilities and community HIV testing sites. Led by lay counselors, 1 group-counseling session and at least 1 individual-counseling session were recommended, and both could happen on the same day as HIV diagnosis, care enrollment, and ART initiation. Adherence counseling support continued thereafter according to patients’ needs. Routine follow-up visits were scheduled at 2, 4, and 12 weeks after ART initiation and every 3 months thereafter. Routine viral load monitoring was performed 6 and 12 months after ART initiation and annually thereafter. Patients with viral loads >1,000 copies/mL received enhanced adherence counseling over 3 months and were switched to second-line ART in case of viral failure (2 consecutive viral load measurements of >1,000 copies/mL) (27). Patients who missed their clinical appointment for medication refills received follow-up by telephone with the possibility of home visits.

### Study design

This is a nested, retrospectively established cohort of adults aged ≥16 years old initiating standard first-line ART under the treat-all programmatic approach in Nhlangano health zone either on the day of facility-based HIV care enrollment (same-day ART) or 1–14 days after HIV care enrollment (early ART), between October 10, 2014, and March 31, 2016. A standard first-line treatment regimen contained a 3-drug combination of lamivudine with tenofovir or zidovudine and efavirenz or nevirapine. A patient was considered enrolled in HIV care and initiated on ART if a paper and/or electronic patient record was created. In this setting, we considered early ART as a relevant comparison group to same-day ART because this was the national policy at the time of the study.

### Analyses and main definitions

Analyses were performed using Stata, version 14.1 (StataCorp LP, College Station, Texas), and R (R Foundation for Statistical Computing, Vienna, Austria). First, baseline characteristics were described with frequency statistics and proportions. The Pearson’s χ^2^ and Mann-Whitney U test were used to compare differences in categorical and continuous variables. We used multiple imputation by chained equations (28) to deal with missing values of the measured pretreatment variables (see Web Table 1, available at https://doi.org/10.1093/aje/kwab032).

**Figure 1:**
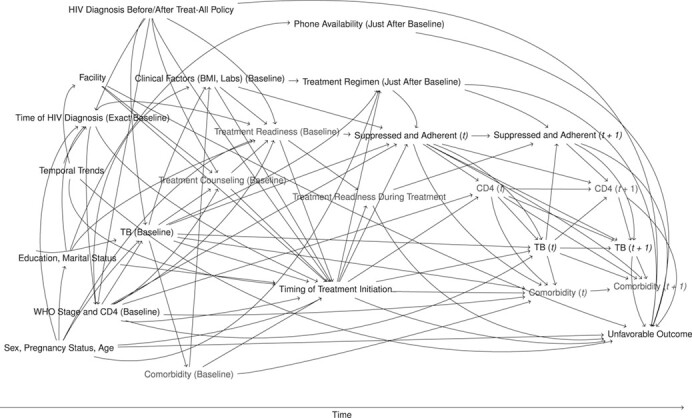
Directed acyclic graph showing structural assumptions about the data-generating process, same-day antiretroviral therapy under treat-all, 2014–2016. Unmeasured variables are shown in gray text. BMI, body mass index; *t*, time; TB, tuberculosis; WHO, World Health Organization.

Second, we assessed predictors of same-day ART initiation compared with early ART by using multivariable Poisson regression models including all variables measured before treatment initiation (listed in Table 1 and Figure 1).

**Table 1:** Baseline Characteristics of Patients Initiated on Antiretroviral Therapy Under Same-Day and Early Protocols, and Predictors of Same-Day Antiretroviral Therapy Initiation, Under the Treat-All Policy, Eswatini, 2014–2016

		**Baseline Characteristics (*n* = 1,328)**					**Predictors of Same-Day ART (*n* = 1,328)**			
**Characteristic**	**% Missing Values**	**Same-Day ART (*n* = 839; 63.2%)**		**Early ART (*n* = 489; 36.8%)**		***P* Value**	**Crude RR**	**95% CI**	**Adjusted RR**	**95% CI**
Year	0.0					<0.001				
2014		117	13.9	88	18.0		1.00	Referent	1.00	Referent
2015		552	65.8	359	73.4		1.06	0.87, 1.30	1.03	0.83, 1.27
2016		170	20.3	42	8.6		1.41	1.11, 1.78	1.20	0.93, 1.55
Timing of HIV diagnosis^a^	0.5					0.064				
Before treat-all		139	16.7	63	12.9		1.00	Referent	1.00	Referent
Under treat-all		694	83.3	426	87.1		0.90	0.75, 1.08	1.18	0.85, 1.65
Facility	0.0					<0.001				
SHC		197	23.5	265	54.2		1.00	Referent	1.00	Referent
PHC 1		62	7.4	41	8.4		1.41	1.06, 1.88	1.45	1.07, 1.97
PHC 2		60	7.2	31	6.3		1.55	1.16, 2.06	1.54	1.15, 2.08
PHC 3		35	4.2	33	6.7		1.21	0.84, 1.73	1.20	0.83, 1.72
PHC 4		88	10.5	14	2.9		2.02	1.57, 2.60	1.96	1.51, 2.54
PHC 5		69	8.2	6	1.2		2.16	1.64, 2.84	2.31	1.73, 3.09
PHC 6		130	15.5	25	5.1		1.97	1.58, 2.45	1.80	1.42, 2.28
PHC 7		165	19.7	39	8.0		1.90	1.54, 2.33	1.84	1.47, 2.30
PHC 8		33	3.9	35	7.2		1.14	0.79, 1.65	1.17	0.80, 1.70
Time from HIV diagnosis to care enrollment	0.5					<0.001				
Same-day		426	51.2	237	48.6		1.00	Referent	1.00	Referent
1–89 days		239	28.7	194	39.8		0.86	0.73, 1.01	1.02	0.87, 1.21
≥90 days		167	20.1	57	11.7		1.16	0.97, 1.38	1.38	1.01, 1.88
Sex/pregnancy	0.6					<0.001				
Men		192	23.0	146	30.0		1.00	0.84, 1.19	1.14	0.94, 1.38
Nonpregnant women		350	41.9	266	54.7		1.00	Referent	1.00	Referent
Pregnant women		293	35.1	74	15.2		1.41	1.20, 1.64	1.37	1.15, 1.62
Age at ART initiation, years	0.0					0.042				
16–24		220	26.2	104	21.3		1.09	0.93, 1.27	1.03	0.86, 1.22
25–49		570	67.9	344	70.3		1.00	Referent	1.00	Referent
≥50		49	5.8	41	8.4		0.87	0.65, 1.17	1.01	0.75, 1.37
Marital status	2.0					0.318				
Married		252	30.8	162	33.5		1.00	Referent	1.00	Referent
Not married		566	69.2	322	66.5		1.05	0.91, 1.22	1.04	0.89, 1.21
Education	16.0					0.037				
None		23	3.2	19	4.7		1.00	Referent	1.00	Referent
Primary		153	21.4	111	27.6		1.03	0.68, 1.55	1.02	0.67, 1.56
Secondary		523	73.2	267	66.4		1.19	0.80, 1.76	1.12	0.74, 1.69
Tertiary		15	2.1	5	1.2		1.36	0.74, 2.50	1.23	0.66, 2.30
CD4 count, cells/mm^3^^b^	3.9					<0.001				
0–100		107	13.4	103	21.6		0.78	0.62, 0.98	0.87	0.68, 1.11
101–200		125	15.6	87	18.2		0.91	0.73, 1.13	0.92	0.73, 1.15
201–350		206	25.8	111	23.3		1.00	Referent	1.00	Referent
351–500		174	21.8	94	19.7		1.00	0.82, 1.22	0.99	0.81, 1.21
≥501		187	23.4	82	17.2		1.07	0.88, 1.30	1.05	0.86, 1.29
WHO clinical stage^b^	0.8					<0.001				
I/II		642	77.2	281	57.9		1.00	Referent	1.00	Referent
III		114	13.7	119	24.5		0.71	0.58, 0.86	0.91	0.74, 1.13
IV		76	9.1	85	17.5		0.68	0.54, 0.86	0.93	0.70, 1.22
Tuberculosis^b^^,^^c^	0.0					<0.001				
No		808	96.3	449	91.8		1.00	Referent	1.00	Referent
Yes		31	3.7	40	8.2		0.68	0.47, 0.97	0.83	0.56, 1.21
BMI^b^^,^^d^	8.8					<0.001				
<18.5		34	4.6	38	8.1		0.85	0.61, 1.18	1.01	0.72, 1.43
18.5–24.9		345	46.5	268	57.1		1.00	Referent	1.00	Referent
≥25.0		363	48.9	163	34.8		1.20	1.04, 1.38	1.10	0.94, 1.28
Hemoglobin^b^, g/dL	24.1					0.063				
≤9		132	21.9	69	17.1		1.12	0.95, 1.32	1.13	0.95, 1.35
≥10		472	78.1	335	82.9		1.00	Referent	1.00	Referent
ALT^b^, U/L	22.5					0.124				
≤42		561	89.8	350	86.6		1.00	Referent	1.00	Referent
≥43		64	10.2	54	13.4		0.90	0.71, 1.13	0.99	0.78, 1.27
Creatinine^b^, μmol/L	17.5					0.113				
≤120		654	98.1	414	96.5		1.00	Referent	1.00	Referent
≥121		13	1.9	15	3.5		0.78	0.47, 1.29	0.79	0.47, 1.33
Telephone availability	1.3					0.121				
No		70	8.5	54	11.1		1.00	Referent	1.00	Referent
Yes		754	91.5	433	88.9		1.13	0.88, 1.44	1.03	0.80, 1.34

Abbreviations: ALT, alanine transaminase; ART, antiretroviral therapy; BMI, body mass index; CI, confidence interval; HIV, human immunodeficiency virus; PHC, primary health-care facility; RR, risk ratio; SHC, secondary health-care facility; TB, tuberculosis; WHO, World Health Organization.

^a^ The HIV-diagnosis timing covariate describes whether HIV-positive diagnosis was established before or during the roll-out of the treat-all policy.

^b^ Baseline clinical and laboratory data were obtained at the time of ART initiation and categorized into normal and pathological.

^c^ A baseline TB case was any incident TB case from 6 months before to 1.5 months after ART initiation.

^d^ BMI = weight (kg)/height (m)^2^.

Third, we emulated a target trial (29–31) of HIV-infected patients aged ≥16 years already initiated on ART within 14 days of facility-based HIV care enrollment to estimate the causal effect (32) of same-day ART (vs. early ART) on the composite unfavorable treatment outcome of death, loss to follow-up (LTFU), viral failure, and treatment switching to a second-line ART in the absence of documented viral failure. Time zero was the date of ART initiation because some captured outcomes (viral failure, treatment switch) could only have happened after ART initiation, and the outcomes of death and LTFU before ART initiation were not well defined (e.g., pretreatment visits were not recorded after care enrollment, which could lead to possible misclassification of deaths as LTFU). Therefore, our target population excluded patients starting treatment >14 days after care enrollment and patients never starting treatment for any reason (including deaths within 14 days of care enrollment).

Viral failure was defined as 2 consecutive viral load measurements >1,000 copies/mL measured at least 5 months after ART initiation and 1.5 months apart. The composite endpoint was chosen to reflect the goals of the treat-all policy and the Joint United Nations Programme on HIV/AIDS 90-90-90 cascade targets of keeping patients on effective ART (virally suppressed) and reducing transmission of HIV. Minimum follow-up time before database closure was 7 months. Patients were censored at the last clinic visit date, when a transfer out was recorded by the clinician, and at database closure (October 31, 2017). LTFU was defined as no-show to the facility for ≥6 months measured at the last clinic visit. Lacking local evidence, no assumptions were made about possible reasons of LTFU such as undocumented deaths, silent transfer out, unstructured treatment interruptions, or actual disengagement from care (33–35).

We summarized our assumptions about the data-generating process in a directed acyclic graph (Figure 1); see Web Appendix 1 for a detailed explanation. Briefly, treatment assignment was based on various factors, including pregnancy, clinician’s preference in each facility, temporal trends, the patient’s perceived readiness and the impact of counseling, and clinical assessment including CD4 count and comorbidities. Timing of treatment initiation might affect the composite outcome in different ways: first, biologically, if treatment delay would affect viral suppression and thus the development of comorbidities and negative outcomes; second, earlier treatment might have a psychological impact on patients. If they do not feel ready for ART and are possibly coerced into treatment, adherence to therapy could be suboptimal and treatment might be interrupted. The directed acyclic graph shows that inclusion of all visualized pretreatment variables, and exclusion of all posttreatment variables (e.g., suppression during follow-up, ART regimen), is sufficient to identify the desired total causal effect (because all back-door paths are blocked and no mediators are being conditioned on) (36). However, because treatment readiness and counseling, as well as some baseline comorbidities (e.g., cryptococcal meningitis), are unmeasured, some remaining unmeasured confounding might persist in our analysis.

Based on the above assumptions, we included all measured pretreatment variables in an adjusted flexible parametric survival analysis (Royston-Parmar models) (37, 38) to estimate the effect of same-day ART initiation on the hazard of the unfavorable outcome. We visualized the results of this model using averaged failure and hazard difference curves to compare the time to the composite unfavorable outcome between same-day and early ART (37, 38). We allowed the effect of same-day ART to vary with respect to time.

Then, we used targeted maximum likelihood estimation (TMLE) (39, 40) to estimate the probability of experiencing the unfavorable outcome 12 months, 18 months, and 24 months after ART initiation under same-day and early ART, and under no censoring, using all measured pretreatment variables. TMLE requires estimation of the expected outcome, treatment assignment, and censoring processes, given the measured covariates. We facilitated this step using extensive super learning to avoid model misspecification (see Web Tables 2 and 3) (41, 42).

**Figure 2:**
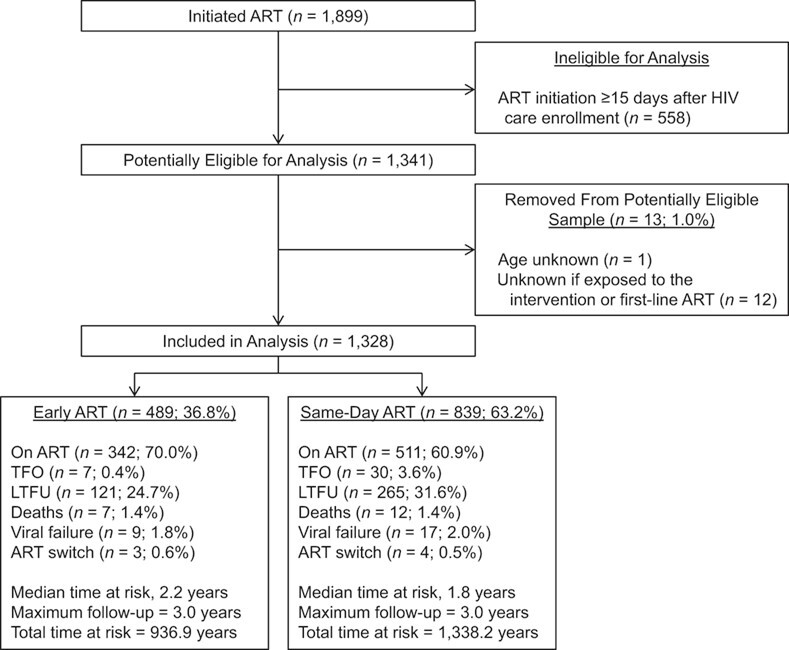
Study flow chart, same-day antiretroviral therapy (ART) under a treat-all policy, Eswatini, 2014–2016. HIV, human immunodeficiency virus; LTFU, loss to follow-up; TFO, transfer out.

**Figure 3:**
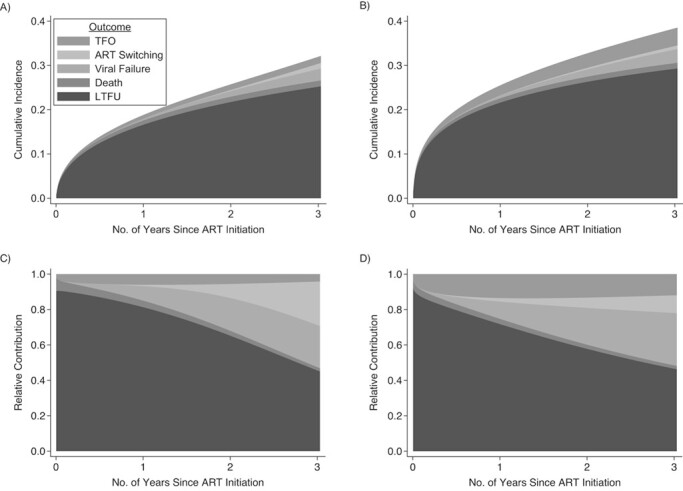
Stacked cause-specific cumulative incidence functions and stacked cause-specific relative contributions to the overall hazard of the outcomes for early versus same-day antiretroviral therapy (ART) under a treat-all policy, Eswatini, 2014–2016. Stacked cause-specific cumulative incidence functions (early ART (A); same-day ART (B)) and stacked cause-specific relative contribution to the overall hazard (early ART (C); same-day ART(D)) of the outcomes of loss to follow-up (LTFU), death, viral failure, treatment switching, and censoring due to transfer out (TFO) for early versus same-day ART. We used the competing risks postestimation command stpm2cif in Stata (StataCorp LP) (59) to estimate the cumulative incidence function for different causes of the outcome (A and B) and the relative contribution to the overall hazard for different causes of the outcome (C and D). The curves are based on a flexible parametric survival model (Royston-Parmar models) (37, 38) using restricted cubic splines. For both interventions, the relative cause-specific contribution of LTFU decreased from approximately 90% at the time of ART initiation to less than half at the end of the observation period (C and D). The cumulative incidence of LTFU was lower for early ART (A) after treatment initiation, but its relative contribution to the outcomes was more pronounced during the first 2 years after treatment initiation when compared with same-day ART (B). Death was rare and similar between both interventions while cumulative transfer out was higher for same-day ART. For both interventions, the relative contribution of viral failure and ART switching to the overall hazard increased rapidly after 6 months since ART initiation (C and D).

Several supplementary analyses were performed. We compared same-day ART with rapid ART initiation defined as ART initiation 1–7 days after HIV care enrollment (rather than early ART) according to World Health Organization recommendations. Then, the composite unfavorable outcome was decomposed to all-cause attrition (death and LTFU combined). Finally, time zero was defined as the date of HIV care enrollment (instead of ART initiation).

### Ethics

This retrospective analysis was nested within a prospective cohort study assessing the feasibility of the treat-all policy (12) and was approved by the Médecins Sans Frontières ethics review board, the Eswatini National Health Research Review Board, and the Human Research Ethics Committee of the University of Cape Town.

## RESULTS

Figure 2 shows the study flow chart. Of 1,899 patients initiating ART, 1,341 (70.6%) started treatment within 14 days after facility-based HIV care enrollment. Thirteen (1.0%) patients were removed from the analysis, because their study eligibility remained unclear. Of 1,328 patients remaining, 839 (63.2%) started ART on the same day as HIV care enrollment.

### Predictors of same-day ART initiation

Table 1 shows baseline characteristics of patients starting ART same-day and early. In multivariable analysis (Table 1), the risk of same-day ART initiation was higher for 6 of 8 primary-care clinics (vs. secondary-care clinic) with adjusted risk ratios ranging from 1.45–2.31, for patients diagnosed ≥90 days before facility-based HIV care enrollment (adjusted risk ratio = 1.38, 95% confidence interval (CI): 1.01, 1.88) versus diagnosed on the same day as HIV care enrollment, and for pregnant women (adjusted risk ratio = 1.37, 95% CI: 1.15, 1.62) versus nonpregnant women.

### Same-day ART initiation

#### Descriptive analyses.

Crude decomposed outcomes are listed in Figure 2, and their decomposed, stacked cause-specific cumulative incidence functions are presented and described in Figure 3. The crude cumulative hazard of remaining on effective first-line ART (not experiencing the composite unfavorable outcome) was lower for same-day ART (vs. early ART) after ART initiation (see Web Figure 1). For same-day ART, it was 72% (95% CI: 68, 74; vs. early ART, 81%, 95% CI: 77, 84) at 1 year and 62% (95% CI: 59, 66; vs. early ART, 69%, 95% CI: 63, 73) at 3 years (see Web Table 5). The likelihood of experiencing the unfavorable outcome was high immediately after ART initiation, with 3.7% (95% CI: 2.3, 5.8) and 8.7% (95% CI: 7.0, 10.8) of patients under early and same-day ART, respectively, never returning to care.

#### Relative impact of same-day ART on the unfavorable outcome.

Multiple imputation of missing values was successful, with good convergence of the imputation algorithm and good other diagnostics (see Web Figure 2 and 3).

**Figure 4:**
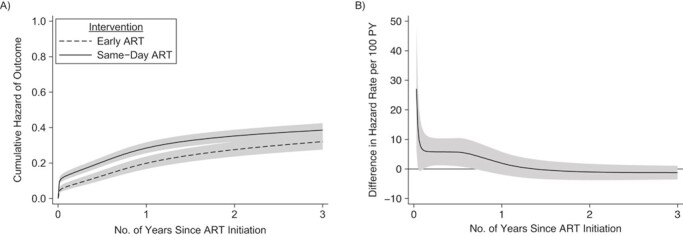
Averaged cumulative hazard (A) and averaged difference in hazard rate (B) of the unfavorable outcome for time from antiretroviral therapy (ART) initiation to unfavorable outcome for patients initiating same-day ART versus early ART, under a treat-all policy, Eswatini, 2014–2016. The adjusted hazard ratio for same-day ART was 1.48 (95% confidence interval: 1.16, 1.89) and varied over time with higher hazard of the unfavorable outcome during the first year of ART. The line at 0 in (B) indicates the reference group (early ART). PY, person-years.

The hazard of the unfavorable treatment outcome was increased for same-day ART by 39% in univariate analysis (crude hazard ratio = 1.39, 95% CI: 1.14, 1.70) and by 48% in multivariable analysis (adjusted hazard ratio = 1.48, 95% CI: 1.16, 1.89), as estimated by the adjusted flexible parametric survival analysis (Figure 4A; see Web Table 4 for the full model). The estimates varied over time, with a higher hazard during the first year after ART initiation and a similar hazard thereafter (Figure 4B).

#### Absolute difference in unfavorable outcomes comparing same-day ART with early ART.

Using TMLE, we estimated that 28.9% (95% CI: 25.4, 32.3) of patients would have experienced an unfavorable outcome after 12 months if they had received same-day ART compared with 21.2% (95% CI: 15.8, 26.6) if they had received early ART, which corresponds to a difference in average treatment effect of 7.7% (95% CI: 1.3, 14.1) and a marginal odds ratio of 1.36 (95% CI: 1.03, 1.81) (see Table 2). Differences between the 2 treatment strategies were also observed for 2 and 3 years of follow-up, although less pronounced than in the first year (see Table 2). Diagnostics of the TMLE approach were satisfactory, with no truncation of estimated probabilities of treatment assignment, small maximum clever covariates, and a broad selection of learning algorithms (see Web Tables 2 and 3).

**Table 2:** Estimated Effect of Same-Day Antiretroviral Therapy Initiation on the Unfavorable Outcome Using Targeted Maximum Likelihood Estimation, Under the Treat-All Policy, Eswatini, 2014–2016^a^

**Time After ART**	**Point Estimate**	**95% CI**
*Average Treatment Effect*		
12 months	0.08	0.01, 0.14
18 months	0.07	0.00, 0.14
24 months	0.06	−0.06, 0.18
*Marginal Odds Ratio*		
12 months	1.36	1.03, 1.81
18 months	1.27	1.00, 1.61
24 months	1.19	0.83, 1.70
*Probability of the Unfavorable Outcome*		
Same-day ART^b^		
12 months	0.29	0.25, 0.32
18 months	0.35	0.31, 0.38
24 months	0.37	0.28, 0.45
Early ART^c^		
12 months	0.21	0.16, 0.27
18 months	0.27	0.22, 0.33
24 months	0.31	0.23, 0.39

Abbreviations: ART, antiretroviral therapy; CI, confidence interval.

^a^ Using a flexible parametric survival model, the adjusted hazard ratio was 1.48 (95% confidence interval: 1.16, 1.89).

^b^ This is the probability of the unfavorable treatment outcome if everybody in the cohort had received same-day ART.

^c^ This is the probability of the unfavorable treatment outcome if everybody in the cohort had received early ART (defined as ART initiation 1–14 days after HIV care enrollment).

#### Supplementary analyses.

Table 3 presents crude and adjusted hazard ratios for different assumptions. Changing time zero to the date of care enrollment, changing the unfavorable composite outcome to all-cause attrition, and comparing same-day ART with rapid ART did not change findings overall, with adjusted hazard ratios ranging from 1.43–1.83.

**Table 3:** Estimates of a Combination of Supplementary Analyses With Different Assumptions, in a Study of Same-Day Antiretroviral Therapy Under the Treat-All Policy, Eswatini, 2014–2016

**Outcomes**	**Crude HR**	**95% CI**	**Adjusted HR**	**95% CI**
*Time Zero: Date of ART Initiation*				
Same-day vs. early ART				
Unfavorable outcome^a^	1.39	1.14, 1.70	1.48	1.16, 1.89
All-cause attrition^b^	1.39	1.13, 1.71	1.47	1.14, 1.88
Same-day vs. rapid ART^c^				
Unfavorable outcome^a^	1.38	1.08, 1.76	1.44	1.08, 1.92
All-cause attrition^b^	1.35	1.05, 1.72	1.43	1.07, 1.92
*Time Zero: Date of HIV Care Enrollment*				
Same-day vs. early ART				
Unfavorable outcome^a^	1.41	1.15, 1.73	1.83	1.41, 2.38
All-cause attrition^b^	1.40	1.14, 1.73	1.67	1.30, 2.16
Same-day vs. rapid ART^c^				
Unfavorable outcome^a^	1.40	1.10, 1.78	1.81	1.33, 2.47
All-cause attrition^b^	1.36	1.06, 1.74	1.80	1.31, 2.47

Abbreviations: ART, antiretroviral therapy; CI, confidence interval; HIV, human immunodeficiency virus; HR, hazard ratio.

^a^ This is the composite unfavorable treatment outcome of death, loss to follow-up, viral failure, and treatment switching to a second-line ART in the absence of viral failure.

^b^ All-cause attrition comprised the outcomes of death and loss to follow-up.

^c^ A total of 1,133 patients initiating ART within 7 days (rapid ART), with 294 (25.9%) within 1–7 days and 839 (74.1%) same-day.

## DISCUSSION

This is, to our knowledge, the first study evaluating faster ART initiation in a routine programmatic HIV-care setting applying the treat-all policy. In patients starting treatment quickly, initiating ART on the day of facility-based HIV care enrollment had inferior treatment outcomes compared with patients starting treatment 1–14 days thereafter or starting treatment within 1–7 days. The estimated effect was accrued during the first year of therapy.

### Interpretation of findings

The main predictors of same-day ART initiation were related to policy and facility factors. Pregnancy in women was associated with increased same-day ART initiation, coinciding with the same-day ART policy under the prevention of mother-to-child transmission “Option B+” approach. Facility-level factors also played a role, with almost all primary-care facilities providing more same-day ART than the secondary-care facility. This might be because primary-care facilities had point-of-care biochemistry, hemoglobin, and CD4 testing available, thus making baseline results available on the same day for treatment decisions, as opposed to the secondary-care facility where results often became available a few days later. Clinicians might have felt more comfortable initiating ART with CD4 cell count and biochemistry known. In addition, the “one stop shop” primary-care clinics provided all HIV services at the same location, whereas HIV testing and care registration were colocated in the secondary-care facility. This required patients diagnosed with HIV in the outpatient department to transfer to the HIV department, thus possibly delaying care registration and ART initiation. More patients might also have had unmeasured comorbidities at the secondary-care facility, necessitating delaying ART initiation.

Patients who knew their HIV-positive status for ≥90 days were more likely to initiate ART on the same day. First, patients might have been transferred in from community HIV testing sites and other facilities. Given more time between testing and care enrollment, they might have come to terms with life-long therapy and therefore been ready to start same-day treatment. Second, treatment interruptions are frequent in routine settings (43), and these patients might have been treatment interrupters reinitiating ART without disclosing prior treatment.

Last, clinical factors as well as social factors, such as level of education and marital status, appeared not to play a major role in quicker ART initiation. This could indicate that same-day ART initiation was driven by facility and health policy factors, as indicated in our analysis, rather than by clinical presentation of the patient, clinicians` considerations, or patients’ preferences.

### Same-day ART

Same-day ART initiation had a higher hazard of the unfavorable treatment outcome than early and rapid ART. Our estimated effect was time-varying, with increased hazard during the first year of treatment and similar hazards thereafter.

We provide several explanations. First, same-day ART might not address patient concerns about expedited ART initiation and might not give enough time to conceptualize lifelong therapy (44–49). This could have contributed to immediate disengagement from care after treatment initiation, with 9% of patients under same-day ART never returning for a follow-up visit.

Second, estimates might be affected by unmeasured confounding. Treatment readiness might predict assignment to the intervention and is also likely associated with the outcome (through the factor of adherence). In addition, we could not measure all possible baseline and time-updated comorbidities that could predict the intervention and the outcome. For instance, cryptococcal meningitis might have been unevenly distributed in the groups and could affect early death and loss to care differently.

Third, the clinical tools used to assess treatment readiness might have been inappropriate to identify patients ready for same-day ART, given that the very same tools were used before same-day ART initiation was an option. Contextualized screening tools for expedited ART initiation adapted to different populations (e.g., pregnant women) and settings might be needed to reliably assess patients’ readiness for same-day treatment. For instance, one randomized trial used a treatment readiness survey to identify patients not ready for same-day ART initiation and excluded them from expedited treatment (16). In addition, training related to expedited counseling protocols and same-day ART for health workers during the early implementation period was lacking, possibly leaving health workers poorly equipped for effective implementation of same-day ART at scale. Last, counseling support after same-day ART initiation might have been deprioritized in this busy public sector setting with competing activities, thus providing insufficient adherence support early during treatment.

#### Findings in context.

The definition of same-day ART differs across studies. Definitions include treatment initiation on the day of HIV diagnosis, day of treatment eligibility, day of HIV care enrollment, or a combination (15–17, 50–52). The same-day ART intervention group often consisted of patients initiating treatment days after the offer of same-day treatment (15, 17, 51), so that studies evaluated the intention to initiate same-day treatment rather than actual same-day treatment initiation (15–17). The offer of same-day ART was often combined with additional interventions (e.g., point-of-care CD4 and biochemistry testing) (53), and restriction of the patient sample to healthier individuals (16) and nonpregnant adults (15–17) might make findings less applicable to routine public sector settings. Streamlining definitions of same-day ART initiation and clarity of what and who is evaluated are warranted.

While same-day ART initiation improves treatment uptake, it might downshift loss to care to the time of treatment (15, 53). Treatment interruptions were already common in routine HIV programs before the introduction of the rapid ART policy (35, 43) and are associated with acquired drug resistance (54). Balancing of patient-level and public health benefits and risks (e.g., unstructured treatment interruptions) is required to make an informed health policy decision.

More emphasis might be needed on a differentiated approach to ART initiation adapted to the patient’s needs, with clinical and programmatic (e.g., logistical) constraints taken into consideration, than on choosing between same-day and rapid/early ART initiation as a blanket approach. In fragile health systems, hasty, low-quality, and possibly coerced ART initiation can occur if HIV programs and funding organizations prioritize achieving targets related to numbers of same-day ART initiations instead of differentiated patient-centered rapid ART initiation.

Importantly, this study did not assess the impact of a policy of same-day ART initiation for all people living with HIV, as this was not feasible in our context (e.g., patients transferred in could not be offered same-day treatment), with the observational study design and available data. Thus, findings are not directly comparable to randomized trials evaluating the offer of same-day treatment to treatment-eligible patients. Our research, however, intends to estimate the risks and benefits of same-day ART initiation for patients with the ability to start treatment early. If there is a causal relationship between same-day ART and unfavorable treatment outcome, then deferral of treatment initiation should be considered for these patients. However, more research into the methods might be required to address questions of frequency, intensity, content, and minimum quality of early adherence support in routine public sector settings.

#### Limitations and strengths.

First, this study assessed outcomes of patients successfully initiated on ART soon after facility-based HIV care enrollment. Restriction allowed the establishment of 2 potentially comparable groups in the context of an observational study design but limits direct comparison with settings where most patients initiate ART 2 weeks after care enrollment. It was beyond the scope of this study to assess outcomes of patients starting treatment late or never, and they might differ in their characteristics and risks for an adverse outcome. By focusing only on one aspect of faster ART initiation, this study did not address the programmatic advantage of same-day ART in reaching patients otherwise being lost to follow-up before treatment. Future studies from the public sector should weigh the benefit of less pretreatment loss with the risk of higher loss early during treatment.

Second, we did not account for loss between the diagnosis of HIV and care enrollment. This might have caused selection bias because only patients successfully linked to facility-based HIV care are considered. Specifically, loss between community-based HIV diagnosis and facility-based enrollment can be high (17, 55), ranging from 10% to more than half in Eswatini (12, 50). Intrafacility linkage in this setting might also be suboptimal; it is estimated to be between 83% and 92% (12, 50, 56). Therefore, findings should not be generalized to predominantly community settings but rather to settings similar to ours, where most HIV diagnosis happens at facility level (57).

Third, patients under same-day ART never returning for refills after treatment initiation could have been silent patient-initiated (undocumented) transfers. The proportion of silent transfers ranges from 5% to 54% in patients documented as LTFU in Africa and is more pronounced in recent and larger treatment cohorts (58). We did not adjust for it because of a weak physical LTFU intervention in place, and the inability of linking patient records to facilities outside the intervention area. Understanding the magnitude of silent transfer under the treat-all policy and whether it differs between same-day and early ART should be further explored to inform health policy.

Fourth, we could not adjust for all possible confounding factors identified in the directed acyclic graph (e.g., comorbidities and treatment readiness), possibly biasing the effect estimate in either direction.

A strength of this study is that we applied different analytical approaches, including state-of-the-art methods (e.g., TMLE), all of which concurred in their main findings. We included a wide range of patients, as found in other HIV programs implementing the treat-all programmatic approach, so findings might be generalizable to similar settings in rural sub-Saharan Africa. This study discussed potential shortfalls in programmatic implementation of the treat-all policy related to contextualized screening tools and training provided, thus drawing attention to the method and quality of implementation.

### Conclusions

Facility and health policy factors were the main predictors of same-day ART initiation. Our data also suggest that same-day ART increased the risk of the composite unfavorable outcome including LTFU. However, LTFU could sometimes relate to silent transfer out; thus, further research about true health outcomes of patients documented as lost to care is urgently needed.

## Supplementary Material

Web_Material_kwab032
