# Supplementary material for: The Impact of Same-Day Antiretroviral Therapy Initiation Under the World Health Organization Treat-All Policy
Source: Am J Epidemiol. 2021 Feb 12;190(8):1519–32. doi: 10.1093/aje/kwab032 (PMC8327202; doi:10.1093/aje/kwab032)
Supplement: Web_Material_kwab032 [file web_material_kwab032.docx]

**Web Material**

**The Impact of Same-Day Antiretroviral Therapy Initiation Under the World Health Organization Treat-All Policy**

Bernhard Kerschberger, Andrew Boulle, Rudo Kuwengwa, Iza Ciglenecki, and Michael Schomaker

Correspondence to Dr. Bernhard Kerschberger, Médecins Sans Frontières, Mantsholo Road 325, Mbabane, Eswatini (e-mail: bernhard.kerschberger@gmail.com)

Author affiliations: Médecins Sans Frontières (Operational Centre Geneva), Mbabane, Eswatini (Bernhard Kerschberger); Centre for Infectious Disease Epidemiology and Research, School of Public Health and Family Medicine, University of Cape Town, Cape Town, South Africa (Bernhard Kerschberger, Andrew Boulle, and Michael Schomaker); Eswatini National AIDS Programme (ENAP), Ministry of Health, Mbabane, Eswatini (Rudo Kuwengwa); Médecins Sans Frontières (Operational Centre Geneva), Geneva, Switzerland (Iza Ciglenecki); and Institute of Public Health, Medical Decision Making and Health Technology Assessment, UMIT - University for Health Sciences, Medical Informatics and Technology, Hall in Tirol, Austria (Michael Schomaker).

**Table of contents:**

- Web Appendix 1: Some explanation of the developed directed acyclic graph (DAG)
- Web Table 1: Pre-treatment variables with missing values, Same-day antiretroviral therapy under Treat-All, 2014–2016.
- Web Table 2: Details and diagnostics^a^ for the TMLE analysis, Same-day antiretroviral therapy under Treat-All, 2014–2016.
- Web Table 3: Details and diagnostics^a^ for the TMLE analysis, Same-day antiretroviral therapy under Treat-All, 2014–2016.
- Web Table 4: Univariate and multivariate analysis of time to unfavourable treatment outcome for patients initiating same-day ART vs early ART, Same-day antiretroviral therapy under Treat-All, 2014–2016.
- Web Table 5: Point estimates of the cumulative hazard of favourable treatment outcome for patients initiating same-day ART vs early ART, Same-day antiretroviral therapy under Treat-All, 2014–2016.
- Web Figure 1: Cumulative hazard of favourable treatment outcome for patients initiating same-day ART vs early ART, Same-day antiretroviral therapy under Treat-All, 2014–2016.
- Web Figure 2: Trace plots of imputed data for all covariates with missing values, Same-day antiretroviral therapy under Treat-All, 2014–2016.
- Web Figure 3: Kernel density plots for imputed CD4 cell count and haemoglobin for all imputed datasets as an example using the *midiagplots* command in Stata, Same-day antiretroviral therapy under Treat-All, 2014–2016.

**Web Appendix 1: Some explanation of the developed directed acyclic graph (DAG)**

Treatment assignment, such as the decision to initiate ART on the same day, was based on the following considerations. Firstly, ART initiation on the day of facility-based HIV care enrolment was policy for pregnant/lactating women and encouraged for other patients in the absence of (presumptive) opportunistic infections. Secondly, same-day ART was practised at the clinician’s discretion based on the clinical and psychological pre-treatment assessment and depended on the patient`s self-perceived readiness, where the clinician’s advice may vary by facility. Thirdly, baseline CD4 cell count may also predict timing of ART initiation (e.g. patients with low CD4 count and no symptoms suggestive of opportunistic infection may initiate ART on the same day, patients with low CD4 cell count and symptoms suggestive of opportunistic infection may delay ART initiation and patients with high CD4 count may initiate immediately or delay). Fourthly, treatment readiness of the patient with regards to psychological preparedness (and not clinical readiness) at baseline could be a main factor predicting same-day ART initiation, as clinicians would not enforce same-day ART if the patient communicated not being ready for it. Fifthly, a patient’s marital status may also affect the decision to start treatment on the same day; married patients may favour deferring the decision on when to start treatment, as they may first wish to consult with their partner. However, higher education level may accelerate initiation, as knowledge of HIV and the benefit of treatment may be increased. Sixthly, the decision to start on the same day may relate to calendar year, as practising clinicians may change advice with increasing experience. Lastly, the time from HIV diagnosis to care enrolment may influence same-day ART, with patients knowing their HIV status for longer possibly being more ready to start treatment immediately. For these reasons, we considered sex, pregnancy status, age, co-morbidities at enrolment, laboratory values at enrolment, education, marital status, temporal trends (calendar year), time of HIV diagnosis, facility, treatment counselling and treatment readiness to be relevant to deciding on the timing of treatment assignment. Consequently, these variables lie on back-door paths from treatment to the outcome.^1^

There are two ways through which timing of ART initiation could affect the composite outcome. The first way is biologically, if treatment delay would affect viral suppression and thus the development of co-morbidities and negative outcomes. Secondly, earlier treatment may have a psychological impact on patients, who, if they did not feel ready for ART and were pushed into treatment, could stop being adherent to their treatment or even be lost to the programme.

Note that all post-treatment variables, which are visualized in the DAG, are mediators on the path from the intervention to the outcome and should thus not be conditioned upon.^1^ However, those variables that determine treatment assignment, as described above, are crucial to block back-door paths from the intervention to the outcome. While we have measured most of these variables, treatment readiness and counselling are unmeasured, as are some baseline comorbidities; this suggests the possibility of some unmeasured confounding.

**Web Table 1: Pre-treatment variables with missing values, Same-day antiretroviral therapy under Treat-All, 2014–2016.**

| Variable | Complete observations | Incomplete observations | |
| --- | --- | --- | --- |
|  |  | n | % |
| CD4 count | 1276 | 52 | 4.1 |
| Body-mass-index | 1207 | 121 | 10.0 |
| Haemoglobin | 1008 | 320 | 31.7 |
| ALT | 1029 | 299 | 29.1 |
| Creatinine | 1096 | 232 | 21.2 |
| Time from HIV diagnosis to care enrolment | 1320 | 8 | 0.6 |
| WHO clinical stage | 1317 | 11 | 0.8 |
| Education | 1116 | 212 | 19.0 |
| Pregnancy status | 1321 | 7 | 0.5 |
| Timing of HIV diagnosis | 1322 | 6 | 0.5 |
| Marital status | 1302 | 26 | 2.0 |
| Phone availability | 1311 | 17 | 1.3 |

*ALT, alanine transaminase; n, number; %, percentage.*

*Footnote: Twelve pre-treatment variables had missing values ranging from 0.5% to 31.7%. The imputation model included all pre-treatment variables as listed in Table 1, viral load follow-up test results and the intervention exposure.*

**Web Table 2: Details and diagnostics^a^ for the TMLE analysis, Same-day antiretroviral therapy under Treat-All, 2014–2016.**

Diagnostics for the product of the estimated probabilities of treatment assignment and censoring; as well as the clever covariate (CC) summary based on the inverse of this product. Ideally, no observations are truncated (i.e. 0% of probabilities are <0.01), the maximum clever covariate is not too high (say <10 or <20), and the mean clever covariate is about 1.

|  | **12 months** | | **24 months** | | **36 months** | |
| --- | --- | --- | --- | --- | --- | --- |
|  | **Same day** | **Early** | **Same day** | **Early** | **Same day** | **Early** |
|  |  |  |  |  |  |  |
| Number uncensored and followed treatment | 814 | 483 | 810 | 482 | 570 | 395 |
| % truncated | 0% | 0% | 0% | 0% | 0% | 0% |
| Mean CC | 0.89 | 0.75 | 0.89 | 0.75 | 0.72 | 0.66 |
| Max. CC | 3.86 | 9.76 | 4.09 | 12.25 | 4.91 | 8.69 |

*^a^The diagnostics were based on a TMLE analysis using the R-package ltmle,^2^ with 10-fold cross validation, a squared loss function (for cross validation), and a truncation level of 0.01, meaning that estimated treatment assignment and censoring probabilities (needed for the targeted update step) would have been truncated if they were lower than 0.01 (although this did not occur). Results averaged over the 10 imputed datasets are reported.*

**Web Table 3:** **Details and diagnostics^a^ for the TMLE analysis, Same-day antiretroviral therapy under Treat-All, 2014–2016.**

Choice of learners: We have used super learning to estimate the i) expected outcome, ii) treatment assignment and iii) censoring processes, given the measured covariates.^3,4^ The learners and screening algorithms used are listed below, together with their average weight in the final prediction algorithm. All learners are from the package super learner (and are described in the respective help file), except “SL.lae” which is from the package MAMI^5^ and implements Lasso Averaging Estimation.^6^ The screening algorithms used are hand-written: screening 5 variables with LASSO^7^ (“lasso”), categorizing variables according to quintiles and using bivariate associations with Cramer’s V^8^ to select the 4 strongest associations (“cramer”) and using Lasso screening where the number of selected variables is chosen by generalized cross validation,^9^ but where “SL.cramer” is used in case this strategy fails (due to numerical reasons); (“lasso2”).

|  |  | **12 months** | | | **24 months** | | | **36 months** | | |
| --- | --- | --- | --- | --- | --- | --- | --- | --- | --- | --- |
|  | *Model* | *Outc.* | *Treat.* | *Cens.* | *Outc.* | *Treat.* | *Cens.* | *Outc.* | *Treat.* | *Cens.* |
|  |  |  |  |  |  |  |  |  |  |  |
| ***Learner*** | ***Screening*** |  |  |  |  |  |  |  |  |  |
| *SL.mean* | *--* | 0.215 | 0.000 | 0.000 | 0.252 | 0.000 | 0.000 | 0.076 | 0.000 | 0.000 |
| *SL.glm* | *--* | 0.051 | 0.216 | 0.000 | 0.000 | 0.212 | 0.000 | 0.020 | 0.205 | 0.283 |
| *SL.bayesglm* | *--* | 0.113 | 0.000 | 0.000 | 0.080 | 0.000 | 0.000 | 0.265 | 0.000 | 0.003 |
| *SL.stepAIC* | *--* | 0.061 | 0.509 | 0.021 | 0.078 | 0.484 | 0.019 | 0.072 | 0.504 | 0.031 |
| *SL.gam* | *--* | 0.000 | 0.000 | 0.002 | 0.000 | 0.000 | 0.019 | 0.002 | 0.000 | 0.072 |
| *SL.knn* | *--* |  | 0.000 | 0.075 |  | 0.000 | 0.075 |  | 0.000 | 0.007 |
| *SL.lae* | *--* | 0.000 | 0.000 | 0.000 | 0.012 | 0.000 | 0.026 | 0.028 | 0.000 | 0.000 |
| *SL.randomForest* | *--* | 0.160 | 0.191 | 0.205 | 0.082 | 0.193 | 0.196 | 0.152 | 0.173 | 0.083 |
| *SL.nnet* | *--* | 0.014 | 0.000 | 0.608 | 0.041 | 0.001 | 0.427 | 0.143 | 0.000 | 0.000 |
| *SL.bayesglm* | *Cramer* | 0.000 | 0.000 | 0.000 | 0.019 | 0.000 | 0.007 | 0.000 | 0.000 | 0.024 |
| *SL.gam* | *Cramer* | 0.000 | 0.000 | 0.000 | 0.014 | 0.000 | 0.005 | 0.029 | 0.000 | 0.225 |
| *SL.step.interaction* | *Cramer* | 0.033 | 0.000 | 0.009 | 0.000 | 0.000 | 0.000 | 0.029 | 0.000 | 0.000 |
| *SL.bayesglm* | *Lasso* | 0.000 | 0.000 | 0.000 | 0.000 | 0.000 | 0.075 | 0.028 | 0.000 | 0.000 |
| *SL.gam* | *Lasso* | 0.000 | 0.000 | 0.000 | 0.000 | 0.000 | 0.058 | 0.000 | 0.000 | 0.018 |
| *SL.step.interaction* | *Lasso* | 0.000 | 0.000 | 0.076 | 0.000 | 0.000 | 0.068 | 0.030 | 0.000 | 0.047 |
| *SL.bayesglm* | *Lasso2* | 0.200 | 0.000 | 0.000 | 0.261 | 0.000 | 0.000 | 0.000 | 0.000 | 0.000 |
| *SL.gam* | *Lasso2* | 0.109 | 0.000 | 0.000 | 0.138 | 0.000 | 0.000 | 0.000 | 0.021 | 0.087 |
| *SL.step.interaction* | *Lasso2* | 0.026 | 0.084 | 0.000 | 0.000 | 0.110 | 0.000 | 0.025 | 0.097 | 0.031 |
| *SL.earth* | *Cramer* | 0.016 | 0.000 | 0.000 | 0.019 | 0.000 | 0.017 | 0.008 | 0.000 | 0.042 |
| *SL.earth* | *Lasso* | 0.000 | 0.000 | 0.003 | 0.003 | 0.000 | 0.006 | 0.093 | 0.000 | 0.048 |

*^a^The diagnostics were based on a TMLE analysis using the R-package ltmle,^2^ with 10-fold cross validation, a squared loss function (for cross validation), and a truncation level of 0.01, meaning that estimated treatment assignment and censoring probabilities (needed for the targeted update step) would have been truncated if they were lower than 0.01 (although this did not occur). Results averaged over the 10 imputed datasets are reported.*

**Web Table 4: Univariate and multivariate analysis of time to unfavourable treatment outcome for patients initiating same-day ART vs early ART, Same-day antiretroviral therapy under Treat-All, 2014–2016.**

| (% missing values) | **Univariate analysis (n=1328)** | | **Multivariate analysis (n=1328)^1^** | |
| --- | --- | --- | --- | --- |
|  | cHR | 95% CI | aHR | 95% CI |
| **Intervention, (0%)** |  |  |  |  |
| Early ART | 1 |  | 1 |  |
| Same-day ART | 1.39 | 1.14, 1.70 | 1.48 | 1.16, 1.89 |
| **Year, (0%)** |  |  |  |  |
| 2014 | 1 |  | 1 |  |
| 2015 | 1.10 | 0.84, 1.43 | 1.12 | 0.84, 1.48 |
| 2016 | 1.27 | 0.91, 1.77 | 1.21 | 0.84, 1.75 |
| **Timing of HIV diagnosis, (0.5%)** |  |  |  |  |
| Pre Treat-All | 1 |  | 1 |  |
| Treat-All | 1.09 | 0.83, 1.41 | 0.76 | 0.47, 1.22 |
| **Facility, (0%)** |  |  |  |  |
| SHC | 1 |  | 1 |  |
| PHC-1 | 1.25 | 0.88, 1.78 | 1.17 | 0.80, 1.72 |
| PHC-2 | 1.32 | 0.92, 1.91 | 1.37 | 0.93, 2.01 |
| PHC-3 | 0.65 | 0.38, 1.10 | 0.65 | 0.38, 1.13 |
| PHC-4 | 0.89 | 0.60, 1.33 | 0.80 | 0.53, 1.23 |
| PHC-5 | 0.68 | 0.41, 1.12 | 0.64 | 0.37, 1.09 |
| PHC-6 | 1.40 | 1.04, 1.89 | 1.41 | 1.01, 1.97 |
| PHC-7 | 1.21 | 0.92, 1.61 | 1.17 | 0.85, 1.61 |
| PHC-8 | 1.02 | 0.65, 1.60 | 1.04 | 0.65, 1.64 |
| **Time from HIV diagnosis to care enrolment, (0.5%)** |  |  |  |  |
| Same-day | 1 |  | 1 |  |
| 1–89 days | 0.92 | 0.75, 1.14 | 1.00 | 0.80, 1.26 |
| ≥90 days | 0.89 | 0.68, 1.17 | 0.73 | 0.46, 1.15 |
| **Sex/pregnancy, (0.6%)** |  |  |  |  |
| Men | 0.91 | 0.71, 1.16 | 0.92 | 0.70, 1.22 |
| Non-pregnant women | 1 |  | 1 |  |
| Pregnant women | 1.30 | 1.05, 1.62 | 1.20 | 0.94, 1.54 |
| **Age at ART initiation, years, (0%)** |  |  |  |  |
| 16 to 24 | 1.44 | 1.17, 1.77 | 1.30 | 1.02, 1.65 |
| 25 to 49 | 1 |  | 1 |  |
| ≥50 | 0.76 | 0.49, 1.18 | 0.88 | 0.56, 1.39 |
| **Marital status, (2.0%)** |  |  |  |  |
| Married | 1 |  | 1 |  |
| Not married | 1.40 | 1.13, 1.74 | 1.28 | 1.02, 1.61 |
| **Education, (16.0%)** |  |  |  |  |
| None | 1 |  | 1 |  |
| Primary | 0.89 | 0.51, 1.56 | 0.91 | 0.51, 1.63 |
| Secondary | 1.09 | 0.64, 1.86 | 0.99 | 0.55, 1.78 |
| Tertiary | 1.04 | 0.41, 2.61 | 0.91 | 0.35, 2.40 |
| **CD4 count, cells/mm^3^, (3.9%)** |  |  |  |  |
| 0 to 100 | 1.31 | 0.97, 1.78 | 1.33 | 0.95, 1.86 |
| 101 to 200 | 0.95 | 0.68, 1.32 | 0.97 | 0.69, 1.37 |
| 201 to 350 | 1 |  | 1 |  |
| 351 to 500 | 1.26 | 0.94, 1.69 | 1.26 | 0.94, 1.69 |
| ≥501 | 1.25 | 0.93, 1.67 | 1.25 | 0.92, 1.68 |
| **WHO clinical stage, (0.8%)** |  |  |  |  |
| I/II | 1 |  | 1 |  |
| III | 0.85 | 0.65, 1.10 | 1.02 | 0.77, 1.36 |
| IV | 1.22 | 0.93, 1.60 | 1.54 | 1.09, 2.18 |
| **Tuberculosis, (0%)** |  |  |  |  |
| No | 1 |  | 1 |  |
| Yes | 0.78 | 0.50, 1.23 | 0.75 | 0.46, 1.21 |
| **BMI, kg/m^2^, (8.8%)** |  |  |  |  |
| <18.5 | 1.29 | 0.87, 1.93 | 1.23 | 0.81, 1.88 |
| 18.5 to 24.9 | 1 |  | 1 |  |
| ≥25 | 0.93 | 0.75, 1.16 | 0.90 | 0.71, 1.15 |
| **Haemoglobin, g/dL, (24.1%)** |  |  |  |  |
| ≤9 | 1.35 | 1.05, 1.73 | 1.21 | 0.93, 1.58 |
| ≥10 | 1 |  | 1 |  |
| **ALT, U/L, (22.5%)** |  |  |  |  |
| ≤42 | 1 |  | 1 |  |
| ≥43 | 0.88 | 0.62, 1.24 | 0.91 | 0.63, 1.30 |
| **Creatinine, µmol/L, 17.5%)** |  |  |  |  |
| ≤120 | 1 |  | 1 |  |
| ≥121 | 1.74 | 1.02, 2.98 | 1.96 | 1.13, 3.38 |
| **Phone availability, (1.3%)** |  |  |  |  |
| No | 1 |  | 1 |  |
| Yes | 0.84 | 0.61, 1.14 | 0.73 | 0.52, 1.02 |

*ALT, alanine transaminase; aHR, adjusted hazard ratio; cHR, crude hazard ration; ART, antiretroviral therapy; BMI, body mass index; n, number; PHC, primary health care facility; SHC, secondary health care facility; WHO, World Health Organization.*

*Footnote: A standard first-line treatment regimen contained 3TC, TDF or AZT, and EFV or NVP. A baseline TB case was any incident TB case between 6 months before and 1.5 months after ART initiation. Baseline clinical and laboratory data were obtained at the time of ART initiation and categorized into normal and pathological. The covariate HIV diagnosis describes whether HIV-positive diagnosis was established before or during the roll-out of the Treat-All policy.*

*^1^Model specifications: The flexible parametric survival model had 3 internal knots (4 degrees of freedom) and 1 internal knot (2 degrees of freedom) for the time-varying covariate intervention exposure.*

**Web Table 5: Point estimates of the cumulative hazard of favourable treatment outcome for patients initiating same-day ART vs early ART, Same-day antiretroviral therapy under Treat-All, 2014–2016.**

| **Time since ART** | **Early ART** | | **Same-day ART** | |
| --- | --- | --- | --- | --- |
|  | **%** | **95% CI** | **%** | **95% CI** |
| **1 day*** | 96 | 94, 98 | 91 | 89, 93 |
| **3 months** | 91 | 88, 93 | 84 | 81, 86 |
| **6 months** | 86 | 83, 89 | 79 | 76, 81 |
| **1 year** | 81 | 77, 84 | 72 | 68, 74 |
| **2 years** | 73 | 69, 77 | 64 | 61, 68 |
| **3 years** | 69 | 63, 73 | 62 | 59, 66 |

** These are patients who initiated ART but never returned for a follow-up visit.*

*ART, antiretroviral therapy.*

*ART, antiretroviral therapy.*

**Web Figure 1:** **Cumulative hazard of favourable treatment outcome for patients initiating same-day ART vs early ART, Same-day antiretroviral therapy under Treat-All, 2014–2016.**

*ALT, alanine transaminase; VL, viral load.*

**Web Figure 2:** **Trace plots of imputed data for all covariates with missing values, Same-day antiretroviral therapy under Treat-All, 2014–2016.**

**Web Figure 3: Kernel density plots for imputed CD4 cell count and haemoglobin for all imputed datasets as an example using the *midiagplots* command in Stata, Same-day antiretroviral therapy under Treat-All, 2014–2016.**

**References**

1. Pearl J. An Introduction to Causal Inference. *Int J Biostat*. 2010;6(2).

2. Lendle SD, Schwab J, Petersen ML, Laan MJ van der. ltmle: An R package implementing targeted minimum loss-based estimation for longitudinal data. *J Stat Softw*. 2017;81(1):1-21.

3. van der Laan MJ, Polley EC, Hubbard AE. Super learner. *Stat Appl Genet Mol Biol*. 2007;6:Article25.

4. van der Laan M, Rose M, Rose E. *Targeted Learning*. Springer International Publishing; 2011.

5. Schomaker M. MAMI: Model Averaging (and Model Selection) after Multiple Imputation - R package Version 0.9.12 2018. http://mami.r-forge.r-project.org. Accessed November 30, 2019.

6. Schomaker M. Shrinkage averaging estimation. *Stat Pap*. 2012;53(4):1015-1034.

7. Tibshirani R. Regression Shrinkage and Selection via the Lasso. *J R Stat Soc Ser B Methodol*. 1996;58(1):267-288.

8. Heumann C, Schomaker M, Shalabh. *Introduction to Statistics and Data Analysis : With Exercises, Solutions and Applications in R*. Springer International Publishing; 2016.

9. Golub GH, Heath M, Wahba G. Generalized Cross-Validation as a Method for Choosing a Good Ridge Parameter. *Technometrics*. 1979;21(2):215-223.
